# Trading-off health safety, civil liberties, and unemployment based on communication strategies: the social dilemma in fighting pandemics

**DOI:** 10.1371/journal.pone.0318541

**Published:** 2025-03-03

**Authors:** Besarta Veseli, Rouven Seifert, Michel Clement, Edlira Shehu

**Affiliations:** 1 Assistant Professor of Marketing, Institute for Marketing, University of Hamburg, Hamburg, Germany; 2 Assistant Professor of Service Management and Digital Transformation, Institute for Marketing & Service Research, University of Rostock, Rostock, Germany; 3 Professor of Marketing and Media, Institute for Marketing, University of Hamburg, Hamburg, Germany; 4 Professor of Digital Marketing, Department of Marketing, University of Groningen, Groningen, Netherlands; University of Siena, ITALY

## Abstract

Crisis management often requires decisions that prioritize the collective good over individual interests. Effective crisis communication strategies can influence individuals’ behavior towards the collective good, preventing negative societal externalities. However, little is known about how these strategies affect individual acceptance of decisions that involve trade-offs between individual and collective interests. We study individual choice behavior regarding maintaining or lifting government-imposed restrictions on private and public life in a referendum setting in the context of the COVID-19 pandemic. Maintaining or lifting the restrictive measures represents a social dilemma that involves trade-offs between civil liberties, health safety, and economic consequences. In three online experiments, we test the impact of communication strategies that focus on health and/or economic factors, as well as risk attribution (i.e. who is at risk by an increase of infections), on individual acceptance of restrictive measures. Results across all experiments show that the majority favors maintaining the COVID-19 measures, indicating that individuals act ethically by trading off individual harm (i.e., restrictions on private and public life) for the prevention of increased societal harm (i.e., infections, deaths). When communication focuses only on health factors, acceptance levels remain robust, regardless of whether the risk is attributed to others, the individual’s group, or the individual. However, when economic factors (i.e., unemployment rates) are included, acceptance of restrictive measures significantly drops. Notably, in an economic-focused communication, attributing risk to the individual’s group increases acceptance such that significantly less individuals vote to lift measures when their group is at higher risk. Overall, these results demonstrate the impact of communication strategies on acceptance of crisis management measures: Our findings have implications for policy makers who design communication strategies to enforce restrictive policies in times of crisis.

## Introduction

Managing large-scale crises involves trade-offs with conflicting individual and collective interests, creating a social dilemma. A *social dilemma* is defined as a conflict situation “between immediate individual self-interest and longer-term collective interests” [1]. To increase prosocial behavior, governments rely on communication strategies that provide citizens with information about the ongoing situation. For example, during the COVID-19 pandemic, various public and private organizations provided daily data on infection rates (e.g., the John Hopkins Corona Resource Center). Typically, this information is intended to influence individual behavior in times of crisis. The COVID-19 pandemic provided a unique opportunity to study crisis communication and individual choice behavior when confronted with alternatives that negatively affect individual interests in favor of the collective.

During the pandemic, governments imposed non-medical measures such as social distancing, contact restrictions, mobility restrictions, and the closure of schools and non-essential businesses [2]. All of these measures aimed to reduce virus transmission, secure national health systems, and limit virus-related deaths. However, these measures also restricted civil liberties and affected economic activities, with negative consequences such as higher unemployment rates. Faced with this critical trade-off between civil liberties, health safety, and the economic consequences of COVID-19 measures, crisis communication became crucial to ensure cooperation and help flatten the curve of new infections.

We study how different communication strategies in crisis situations with conflicting individual and societal interests affect an individuals’ acceptance of governmental restrictions. We specifically test individuals’ responses to the communication of health (i.e., infection rates, death rates) and economic factors (i.e., unemployment rates) in combination with different scenarios of risk attribution (i.e., who is at risk from an increase in infections) on their preferences for maintaining or lifting restrictions on private and public life during large-scale crises, including lockdowns.

Despite the growing body of research on the acceptance of COVID-19 measures, empirical evidence about the impact of specific communication strategies on the acceptance of restrictive measures is lacking. A first stream of literature measures acceptance of COVID-19 measures using survey methods [e.g., 3–7]. Other studies use discrete choice experiments to assess relative preferences for different COVID-19 measures (and/or outcome scenarios) in various hypothetical scenarios with a wide range of choice sets [8–13]. Using within-subjects designs might lead to higher hypothetical bias [e.g., as shown in a meta-analysis on measuring willingness to pay [14]. Furthermore, these studies were conducted during the later stages of the pandemic [e.g., 10–12], when individuals may have demonstrated significantly different behavioral patterns compared to the early stages, where collective cooperation was crucial in preventing the spread of infection and averting a global outbreak. Consequently, insights on early-stage individual response to restrictions are scarce. We address this gap by designing three online experiments with trade-off decisions in a referendum setting, where we measure individuals’ voting behavior. Notably, our experiments were conducted during the first lockdown in the first wave of the pandemic while all COVID-19 measures in Germany were still in place (i.e., border closures, closures of schools and non-essential businesses, social distancing requirements, enforcement of mask-wearing, and public gatherings ban [2]. Thus, the scenarios have high external validity because individuals were affected by both health and economic risks in real life at the time that we collected the data, which should reduce a potential hypothetical bias.

Our experimental designs mimic a real-world democratic vote during the first lockdown in Germany and aim to provide behavioral insights into the influence of different communication strategies on voting behavior, where we investigate three communication strategies that have not been analyzed in existing research.

We conducted three online experiments (n1 =  866, n2 =  817, n3 =  1,564) in Germany. The online experiments were conducted from April 22, 2020 to May 04, 2020. We vary the focus of the communication strategies in each of the experiments and manipulate the increase (decrease) in health and/or economic consequences of maintaining or lifting COVID-19 measures: Specifically, we communicate the level of infection rates in Experiment 1, death rates in Experiment 2, and both infection rates and unemployment rates in Experiment 3. Moreover, we manipulated four levels of individual risk attribution (i.e., who is endangered by an increase in infections) in each experiment to analyze a potential moderation. Participants stated acceptance by voting in a referendum setting to maintain or lift the active COVID-19 measures in each experiment.

Our results show that across all three experiments, individuals perceive the trade-off decision to be very strong: Specifically, participants perceive both the communicated threats from lifting COVID-19 measures and the restriction of civil liberties from maintaining COVID-19 measures as substantial. However, despite the social dilemma of restricting civil liberties associated with these measures, acceptance is high as health threats decrease. The results underline that people act ethically when trading-off individual harm (i.e., restrictions on private and public life) for the prevention of increased societal harm (i.e., infections, deaths). If communication only focuses on health aspects like infection and death rates, the results of Experiments 1 and 2 show that they generally remain robust despite various risk attributions. This means that acceptance levels do not significantly change when the risk is attributed to other people versus the participant’s own group, or versus the participant alone. Risk attribution does not have a significant impact on acceptance levels, nor does it encourage opportunistic behavior, except in Experiment 2 where more people voted to lift the measures when their own group was immune than when only they alone were immune. However, a communication strategy focused on economic consequences (Experiment 3), measured by higher unemployment rates as a result of maintaining COVID-19 measures, negatively impacts acceptance. Meaning, significantly more people vote for lifting the measures in Experiment 3 compared to when solely health-related consequences are communicated (Experiments 1 and 2). We also find that when both health-related and economic consequences are communicated (Experiment 3), risk attribution to one’s group compared to others increases acceptance, such that significantly less participants vote to lift the measures when their group is more at risk compared to others. Overall, these results demonstrate the impact of communication strategies on acceptance of crisis management measures: A stronger focus on economic consequences results in less acceptance of the restriction of liberties, whereas a focus on health factors (such as infection or death rates) increases acceptance.

Our findings contribute to the social dilemma research [e.g., 1], particularly to the literature on applied approaches to social dilemmas, which focus on designing practical solutions and strategies to address real-world cooperative challenges. We show the importance of three different communication strategies, that have not been analyzed in former research, on individual cooperation during social dilemmas. In addition, this work adds new insights to the literature on ethical behavior and cooperation during crises [e.g., 15]. Our work also contributes to the COVID-19 literature, particularly to the literature on the acceptance of COVID-19 measures [e.g., 3,4,8,9,12,13]. Our findings provide implications for policy makers when confronted with a social crisis.

## Study framework

Social dilemmas involve a conflict between individual and collective interests in a social situation and suggest that in some situation individuals may act in their own interests, even if this has negative consequences for the common good [1,16]. Consequently, members of a group are faced with the choice of either cooperating for the common good or defecting for self-interest. Social dilemmas can arise in many cases such as in consumer boycotts [e.g., 17], pro-environmental behavior [e.g., 16,18], or the introduction of new technologies, such as self-driving cars and the attribution of responsibility in the event of an accident [19]. In the case of the COVID-19 pandemic, individuals’ civil liberties were severely restricted by various COVID-19 measures [e.g., mobility restrictions, social distancing, ban on public gatherings; 2], particularly, during lockdown periods. These measures are typically based on the notion that COVID-19 is highly contagious and poses a threat to both personal well-being and the well-being of others, following the long-term collective interest to prevent infection rates and, ultimately, deaths.

**Fig 1:**
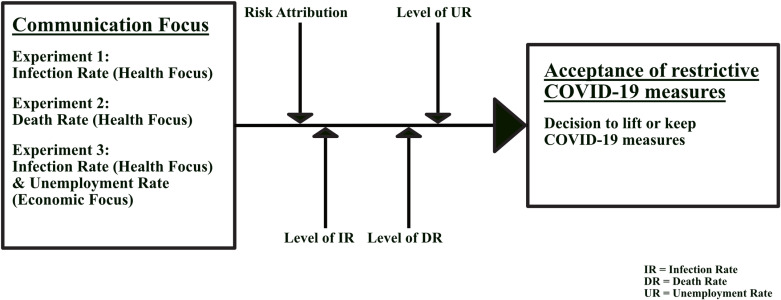
Research framework.

We investigate the impact of communication strategies related to trade-offs in the COVID-19 social dilemma, as illustrated in Fig 1. We focus on three different communication strategies which may influence individuals’ acceptance of restrictive policies (i.e., COVID-19 measures). The communication strategies relate to the communication of health threats (i.e., infection rate, death rate) and economic threats (i.e., unemployment rate) as consequences of lifting or maintaining the restrictive COVID-19 measures.

At the onset of the COVID-19 pandemic, media outlets like *The New York Times*, and government-owned institutions, like the *Robert Koch Institute* provided information on the evolution of the outbreak, monitoring different numbers and rates. In particular, the number of new COVID-19 infections, and the number of deaths were important indicators of the pandemic spread [20,21]. Both metrics can be used in crisis communication to stimulate people to act prosocially by creating psychological affect through communication [22]. However, people’s responses to COVID-19-related metrics may vary with some metrics being more efficient in crisis communication than others. The availability heuristic [23] suggests that death rates may have more associative connections (e.g., more images) compared to infections, making them more likely to promote prosocial behavior (e.g., accepting restrictive measures). For example, high media coverage of COVID-19-related deaths may increase individuals’ vote in favor of restrictive measures. However, daily reports of rising infection rates by media outlets and public institutions can also lead to tangible perceptions of threat. Furthermore, an illusion of control over the situation [24] may influence the acceptance of restrictive measures. As long as people expect to be able to cope with a COVID-19 infection, they will be less influenced by the infection rate, while being susceptible to a COVID-19-related death will appear less controllable and increase the acceptance of restrictive measures. This notion is also supported by considerations of cognitive appraisal formulated in stress and coping theory [25], that individuals can more easily categorize a COVID-19 infection (associated with infection rates) as irrelevant compared to a potential COVID-19-related death (associated with death rates), which feels more dangerous.

In addition, economic consequences such as higher unemployment rates as a result of COVID-19 measures may influence individuals’ votes for lifting or maintaining restrictive measures. Especially lockdowns can have harmful short- and long-term implications for the economy and individuals [26–28]. Communicating high unemployment rates can evoke scenarios of economic insecurity and financial hardship for households, leading to a significant perception of neglecting basic need for (financial) autonomy [29]. Thus, the tangible economic threats posed by communicated unemployment rates may outweigh the more abstract or distant risks associated with infection rates, when communicated together. This may encourage more individuals to vote in favor of lifting restrictive measures.

We further argue that the effect of communication strategies on acceptance of restrictive COVID-19 measures is moderated by risk attribution. Specifically, individuals assess their own and group’s (i.e., their closest relatives) probability of an infection or COVID-19-related death, when voting in favor or against restrictive measures. Especially a higher perceived infection or death probability can be crucial in accepting restrictive COVID-19-related measures [30]. Further, emotional proximity to individuals who are susceptible to a COVID-19-related infection or death may increase the response in favor of accepting measures [31]. The influence of risk attribution becomes especially important with increasing immunization, for instance, an individual could vote for lifting restrictive measures if (currently) immune to a COVID-19 infection. Overall, the COVID-19 pandemic provided a setting with high external validity for studying these trade-off decisions because individuals were in fact in a lockdown and facing potentially harmful consequences on either side of the trade-off. We next describe our experimental settings.

## Materials and methods

Table 1 provides an overview of our three empirical studies. First, we aim to test the trade-off between the civil right to freedom and collective health safety in two experiments. We capture health safety by communicating infection rates in Experiment 1 and death rates in Experiment 2. Next, we test the tradeoff between the civil right to freedom and health safety in combination with economic consequences in Experiment 3.

**Table 1:** Overview of studies.

Study	N	Experimental Factors			
		Risk Attribution	Infection Rate	Death Rate	Unemployment Rate
			baseline = 1.5	baseline = 7.5	baseline = 4
1	866	◦individual at risk	3	n/a	n/a
		◦group at risk			
		◦other than individual at risk	6		
		◦other than group at risk			
2	817	◦individual at risk		15	
		◦group at risk			
		◦other than individual at risk		25	
		◦other than group at risk			
3	1,564	◦individual at risk	3		8
		◦group at risk			
		◦other than individual at risk	6		16
		◦other than group at risk			

The Dean of Research from the Business School of the University of Hamburg reviewed and approved this research proposal with respect to ethics before the data was collected in April 2020. All surveys were conducted via Respondi (now Bilendi) – a professional market research company. The company provides an online access panel. Respondents need to agree to participate in surveys with Respondi (written participant consent). Each survey is reviewed by the company before it is distributed online to ensure consent. Thus, the market research firm manages the written participant consent. This consent procedure was approved by the Dean of Research and the ethics committee of the University of Hamburg.

## Study design

All three online experiments were conducted from April 22, 2020 to May 04, 2020 during the first wave of the pandemic and before any measures in Germany were lifted with active restrictions on private and public life. All experiments were based on a demographically representative sample of the German population (n1 =  866, n2 =  817, n3 =  1,564). For all studies we used a between-subjects design with different conditions. Participants were randomly assigned to one of the conditions. We manipulated infection rates in Experiments 1 and 3, death rates in Experiment 2, and unemployment rates in Experiment 3. All manipulations were on two levels (e.g., high and low level of infection rates) as a result of the individual choice (e.g., lifting the measures). In all three experiments, we further manipulated four levels of risk attribution to investigate whether the decision depends on risk attribution (i.e., who is endangered by an increase in infections). In total, we ended up with 4 (risk attribution) x 2 (infection rate) in Experiment 1, and 4 (risk attribution) x 2 (death rate) in Experiment 2, and 4 (risk attribution) x 2 (infection rate) x 2 (unemployment rate) in Experiment 3 (see Table 1 for the experimental factors and corresponding levels).

## Procedure

At the beginning of the online experiments, participants provided demographic information, as well as COVID-19-related information. After that, participants were shown a baseline level of infection rate, death rate, or unemployment rate, respectively. All baseline numbers represented approximately the infection rate in Germany at that time. Participants were also told who was at risk, that is, whether they themselves or others were susceptible to infection, depending on the experimental condition (Table 1, column 3). Next, we presented a national referendum in which participants were asked to vote to either maintain restrictions on private and public life, or lift the active COVID-19 measures. Both decisions had ultimate consequences on infection rates, death rates, and unemployment rates (e.g., a higher rate of infections, but preserving the baseline level of unemployment). See Supporting Information A for the exact wording of all experimental stimuli and a detailed description of the procedure.

## Measurements

Table 1 provides an overview of the manipulations in the different experimental conditions. In all experimental conditions, we defined a baseline which represents the current pandemic situation in Germany reflected in the specific infection rate, death rate, and unemployment rate. In Experiment 1, we defined the infection rate as the average number of individuals who are infected by an infectious person. We manipulated infection rate with an increase to the baseline by 3 (6) people were infected per infected (baseline: 1.5). In Experiment 2, we defined the death rate as the average number of individuals who died by an infected person. The level of death rates was manipulated with an increase to the baseline by 15% (25%), as a result of lifting the measures (baseline: 7.5%). In Experiment 3, we defined the unemployment rate as the average number of people who became unemployed related to the measures. We manipulated the increase in unemployment rates compared to the baseline by 8% (16%) as a result of maintaining the measures (baseline: 4%). We further manipulated risk attribution across four levels: individual at risk, group at risk (including the individual and their closest relatives), other than individual at risk (i.e., the individual is not personally susceptible to infection), and other than group at risk (i.e., the individual’s group is not personally at risk for infection). For example, participants in the condition “individual at risk” were informed that they have not been infected before and are susceptible to a corona virus infection. See S1 Table in Supporting Information A for the exact wording of the risk attribution stimuli.

We surveyed decision behavior in the referendum with different items. Supporting Information B provides detailed descriptive statistics. Participants were asked to report how difficult it was for them to decide on a 7-point-scale (“How difficult or easy was it for you to decide on a scenario in the referendum?” 1 =  very easy, 7 =  very difficult) and how confident they were with their decision on a 7-point-scale (“How certain or uncertain are you about your decision in the referendum?” 1 =  very uncertain, 7 =  very certain). We further measured perceived threat on a 7-point-scale with four items (“If I vote to lift the measures, I put others at risk/others are put at risk/I will harm others/others are harmed;” α = .93, 1 =  strongly disagree, 7 =  strongly agree) and perceived restriction of civil liberties on a 7-point-scale with four items (“If I vote to maintain the measures, I am restricted in my freedom/others are restricted in their freedom/public and private life is restricted for me/public and private life is restricted for others;” α = .93, 1 =  strongly disagree, 7 =  strongly agree) as voting outcomes.

Finally, participants were asked to state when they expected to be able to go back to their daily life on a scale from 1 =  in the next two weeks, to 7 =  in over one year, and to what extend they felt that their daily life is currently, under the active COVID-19 measures, restricted on a scale from 1 =  not restricted at all, to 7 =  extremely restricted.

## Results

### Perceived trade-off

**Fig 2:**
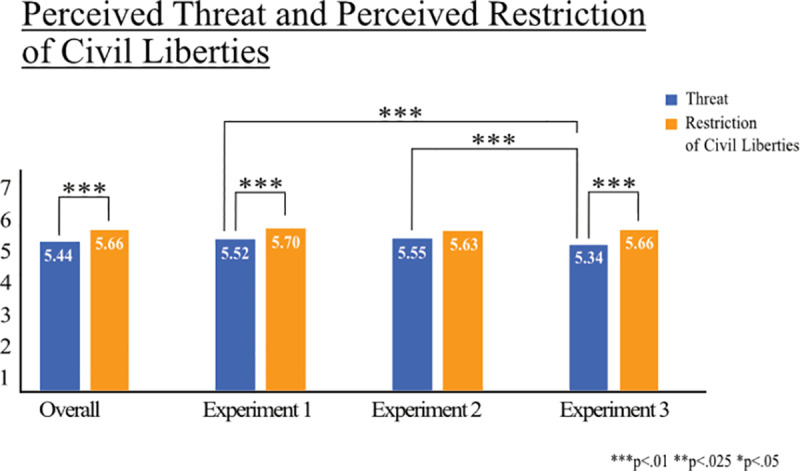
Perceived threat of lifting COVID-19 measures and perceived restriction of liberties if maintaining COVID-19 measures. Note: Comparison of reported mean values of perceived threat when lifting the COVID-19 measures and perceived restriction on civil liberties when maintaining the measures, overall and for each experiment. Perceived threat is measured on a 7-point-scale with four items (“If I vote to lift the measures, I put others at risk/others are put at risk/I will harm others/others are harmed;” 1 = strongly disagree, 7 = strongly agree). Perceived restriction of civil liberties is measured on a 7-point-scale with four items (“If I vote to maintain the measures, I am restricted in my freedom/others are restricted in their freedom/public and private life is restricted for me/public and private life is restricted for others;” 1 = strongly disagree, 7 = strongly agree). Significant differences between values are marked.

We first analyze how strong the trade-off was perceived by participants. We compare the mean values of the perceived threat of lifting measures and the perceived civil liberties restriction if the measures are kept. We make this comparison for each experiment and between experiments (Fig 2).

Overall, participants perceived the consequences of lifting the measures to be threatening to them and others (M =  5.44, SD =  1.42), but felt that civil liberties were more restricted when the measures were in place (M =  5.66, SD =  1.30, t(3246) =  6.84, *p* = *.*000; Fig 2). Perceptions of civil liberty restrictions did not significantly differ across experiments (Experiment 1: M =  5.70, SD =  1.36; Experiment 2: M =  5.63, SD =  1.31; Experiment 3: M =  5.66, SD =  1.23, F(2,3244) =  0.56, *p* = .574). However, lifting the measures was perceived more threatening in Experiment 1 (M =  5.52, SD =  1.41, t(2428) =  2.83, *p* = .005) and Experiment 2 (M =  5.55, SD =  1.35, t(2379) =  3.40, *p* = .000) compared to Experiment 3 (M =  5.34, SD =  1.45). We conclude that the consequences of unemployment–which were part of Experiment 3 but not Experiments 1 and 2 attenuate the perceived threat of lifting the measures.

The perceived trade-off between health safety and restrictions on civil liberties is strongest in Experiment 2 as reported values on both perceived threat (i.e., increase of death rate within risk groups) of lifting measures (M =  5.55, SD =  1.35) and restrictions on civil liberties are high and statistically indifferent (M =  5.63, SD =  1.31, t(816) =  -1.26, *p* = .208; Fig 2). However, in Experiments 1 and 3, the restriction of civil liberties is perceived to be significantly greater compared to the perceived threat (Experiment 1: M =  5.70, SD =  1.36 vs M =  5.52, SD =  1.41, t(865) =  2.89, *p* = .004; Experiment 3: M =  5.66, SD =  1.23 vs M =  5.34, SD =  1.45, t(1563) =  6.72, *p* = .000; Fig 2).

### Perceived decision difficulty and perceived decision confidence

**Fig 3:**
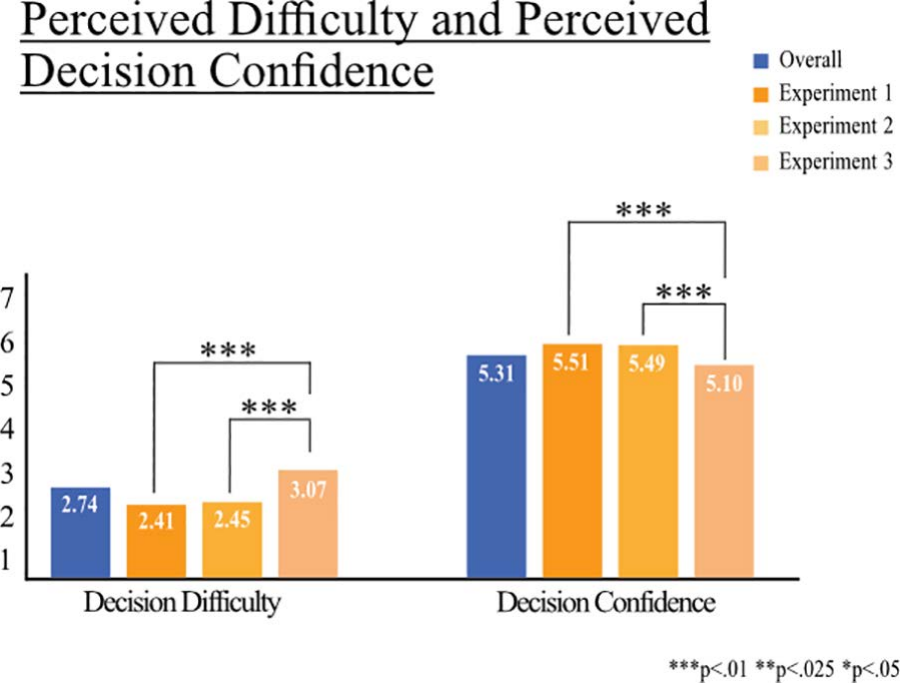
Perceived difficulty making decision and confidence in decision. Note: Reported mean values of perceived difficulty in deciding between lifting or maintaining the COVID-19 measures and perceived confidence in decision, overall and for each experiment. Perceived difficulty is measured on a 7-point-scale (“How difficult or easy was it for you to decide on a scenario in the referendum?” 1 = very easy, 7 = very difficult). Perceived confidence is measured on a 7-point-scale (“How certain or uncertain are you about your decision in the referendum?” 1 = very uncertain, 7 = very certain). Significant differences between values are marked.

We compare mean values of perceived difficulty and perceived confidence in the decision between experiments. Overall, across all experiments, we find that participants had little difficulty making the trade-off decision (M =  2.74, SD =  1.76) and are rather confident in their decision (M =  5.31, SD =  1.61; Fig 3). When comparing Experiment 1 to Experiment 2, we find no statistical difference in perceived decision difficulty (M =  2.41, SD =  1.62 vs M =  2.45, SD =  1.66, t(1681) =  -.49, *p* = .622) nor in decision confidence (M =  5.51, SD =  1.57 vs M =  5.49, SD =  1.58, t(1681) =  0.25, *p* = .800). However, in Experiment 3 where we also vary the unemployment rate, participants have significantly more difficulty making the decision (M =  3.07, SD =  1.83) compared to Experiment 1 (M =  2.41, SD =  1.62, t(2428) =  8.88, *p* = .000) and Experiment 2 (M =  2.45, SD =  1.66, t(2379) =  8.12, *p* = .000). Moreover, participants in Experiment 3 are significantly less confident in their decision (M =  5.10, SD =  1.61) compared to Experiment 1 (M =  5.51, SD =  1.57, t(2428) =  -6.13, *p* = .000) and Experiment 2 (M =  5.49, SD =  1.58, t(2379) =  -5.72, *p* = .000; Fig 3).

### Decision behavior towards restrictions on private and public life

**Fig 4:**
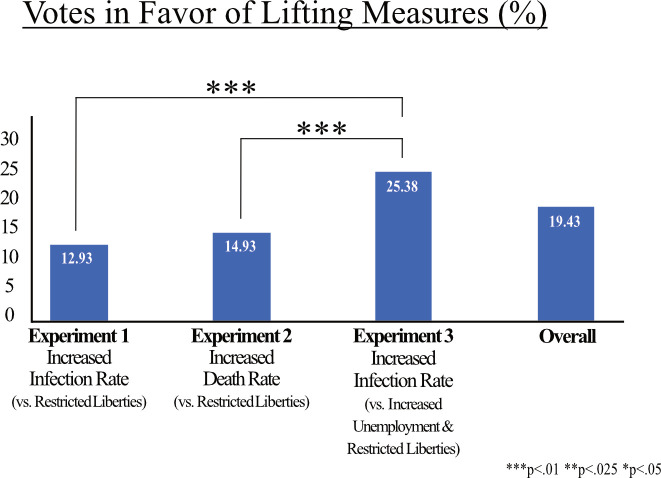
Votes in favor of lifting COVID-19 measures. Note: Proportion of votes in favor of lifting measures, considering the presented trade-off. Significant differences between experiments are marked.

We analyze the individual choice behavior in the COVID-19 social dilemma by comparing the proportion of votes in favor of lifting the active COVID-19 measures between experiments and within experiments. Generally, the majority of participants decided not to lift the measures across all settings, that is, 19.43% vote in favor of lifting the measures (Fig 4). In Experiment 1, which communicates the increase of infection rates as consequence of lifting the measures, 12.93% of the participants voted to lift the measures. We find a slightly higher number of participants (14.93%) favoring to lift the measures in Experiment 2, which communicates death rates within risk groups. However, in Experiment 3, when participants are additionally confronted with trading-off higher unemployment rates versus health risks (i.e., increase of infection rate), the votes in favor of lifting the measures substantially increase to 25.38% compared to both Experiment 1 (*z* =  7.22, *p* = .000) and Experiment 2 (*z* =  5.86, *p* = .000; Fig 4).

**Fig 5:**
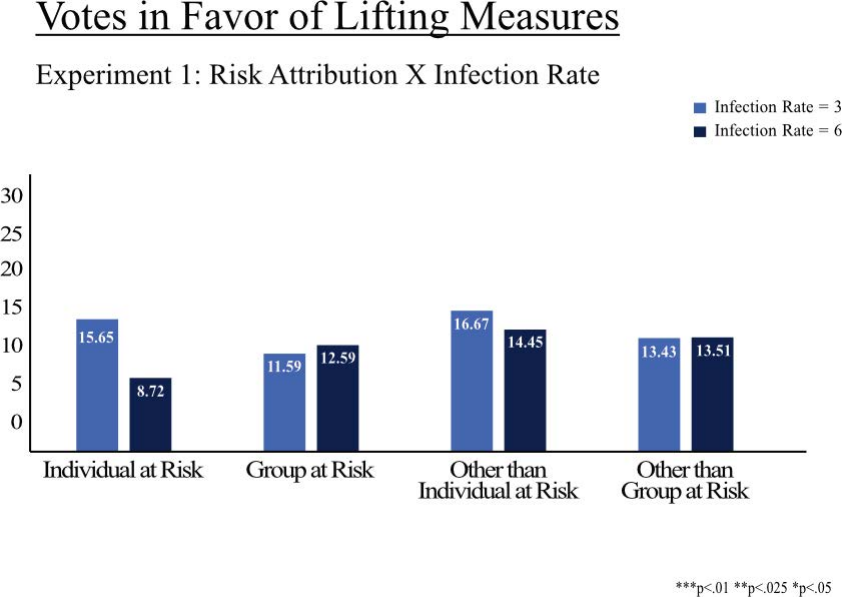
Votes in favor of lifting COVID-19 measures (Experiment 1). Note: Proportion of votes in favor of lifting measures, considering the presented trade-off. There are no significant differences between experimental groups on the significance level of 5%.

**Fig 6:**
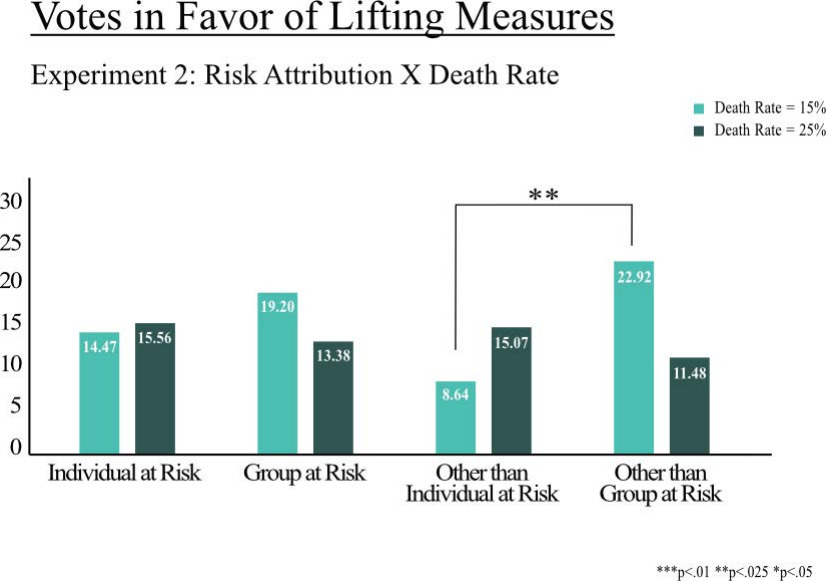
Votes in favor of lifting COVID-19 measures (Experiment 2). Note: Proportion of votes in favor of lifting measures, considering the presented trade-off. Significant differences between experiments are marked.

Fig 5 and Fig 6 show the results of Experiments 1 and 2 across the three experimental factors (1) risk attribution, (2) infection rate, and (3) death rate within risk groups. First, χ^2^-tests were performed to examine the relationship between the proportion of votes in favor of lifting the measures and the experimental factors. For Experiment 1, no significant relationship was found between the outcome of the votes and risk attribution χ^2^ (3, N =  866) =  1.200, *p* = .753) nor infection rate χ^2^ (1, N =  866) =  1.002, *p* = .317). A robustness estimation via logistic regressions also shows no significant relationship between the outcome and the experimental factors (see Supporting Information C.) We also do not find a significant relationship between the outcome of votes and the experimental factors in Experiment 2 (risk attribution: χ^2^ (3, N =  817) =  1.780, *p* = .619; death rate: χ^2^ (1, N =  817) =  0.439, *p* = .508). However, two-sample tests of proportions between the experimental groups in Experiment 2 show that more participants vote in favor of lifting the measures (22.91%) when those outside of their group are at risk (i.e., group is immune) compared to when those other than the participant is at risk (i.e., participant is immune; 8.6%, *z* =  2.262, *p* = .024), but only for lower levels of increased death rate (Fig 6). This finding is also confirmed by a logistic regression that includes interaction effects between risk attribution and the level of death rate. The probability to vote for lifting measures decreases by 62% when people other than the participant are at risk compared to those outside the participant’s group at the lower level of death rate (Supporting Information D). We do not find significant interactions between risk attribution and the level of infection rate in Experiment 1 (Supporting Information C).

In Experiment 3, we conducted a logistic regression to analyze the effects of risk attribution, level of infection rate, and level of unemployment rate on the decision to lift COVID-19 measures (i.e., outcome variable =  1 indicates the decision to lift measures). We find a negative effect of risk attribution on the decision to lift the restrictive measures when a participant’s group is at risk compared to others (odds ratio = .66, *p* = .022; Table 2, Model 1), that is, the probability to decide to lift measures decreases by 34%. The estimated odds ratios for the other levels of risk attribution are not significant (individual at risk:.81, *p* = .230; other than individual at risk:.96, *p* = .809, Table 2, Model 1). Moreover, results show a positive effect of the level of unemployment rate (odds ratio =  1.25, *p* = .054) on the decision to lift measures, that is, an increase to the higher level of unemployment (UR =  16) leads to an increased probability to lift the measures by 25%, compared to a lower-level increase (UR =  8). However, there is no significant effect of the level of infection rate (odds ratio = .86, *p* = .198). We estimated a second logistic regression model which included two COVID-19-related covariates: expected return to daily life and current restriction of daily life. Results show a significant positive effect of the level of unemployment rate (odds ratio =  1.34, *p* = .016; Table 2, Model 2) on the decision to lift the measures, that is, the probability to vote for lifting the COVID-19 measures increases by 34% when the unemployment rate increases to a higher level (UR =  16%) compared to a lower-level increase (UR =  8). On the contrary, acceptance decreases when a participant’s group is at risk compared to others (odds ratio = .67, *p* = .032, Table 2, Model 2). As in Model 1, we find no significant effect for the other levels of risk attribution (individual at risk:.79, *p* = .184; other than individual at risk:.88, *p* = .514), or the level of infection rate (odds ratio = .83, *p* = .122). Additionally, the COVID-19-related covariates significantly drive the probability to decide to lift the restrictions on private and public life (Table 2, Model 2). The probability of voting to lift the COVID-19 measures is reduced by 20% with every point of increase on the expected return to daily life scale (i.e., from 1 =  in two weeks to 7 =  in more than one year). In contrast, the odds for deciding to lift the COVID-19 measures are 1.40 with every point of increase on the perceived current restriction of daily life scale (i.e., from 1 =  not restricted at all to 7 =  extremely restricted), so the probability of lifting the restrictions on private and public life increases by 40%.

**Table 2:** Logistic regression results for the decision to lift measures (Experiment 3).

	Model 1			Model 2		
	Odds Ratio	se	*p*	Odds Ratio	se	*p*
**Risk Attribution**						
Individual at Risk	.81	.142	.230	.79	.142	.184
Group at Risk	.66	.119	.022	.67	.124	.032
Other than Individual at Risk	.96	.179	.809	.88	.171	.514
Other than Group at Risk	*serves as reference group*			*serves as reference group*		
**Infection Rate** (IR = 6, baseline = 3)	.86	.174	.198	.83	.100	.122
**Unemployment Rate** (UR = 16, baseline = 8)	1.25	.117	.054	1.34	.162	.016
**Expected_Return DailyLife**				.80	.034	.000
**Current_Restriction DailyLife**				1.40	.059	.000
**Constant**	.40	.066	.000	.22	.073	.000
N	1,564			1,564		

Note: The outcome variable is binary, where an outcome of 1 indicates the decision to lift measures, while 0 indicates maintaining them. The odds ratios represent the probability of deciding to lift measures compared to the reference group or baseline condition. The reference group for the “Risk Attribution” variable is “Other than Group at Risk.” For the binary variables “Infection Rate” and “Unemployment Rate,” a value of 1 indicates the higher level (IR = 6 and UR = 16, respectively), with the baseline being IR = 3 and UR = 8. The “Expected_Return DailyLife” is measured on a scale from 1 (in the next two weeks) to 7 (in over one year), and “Current_Restriction DailyLife” is measured on a scale from 1 (not restricted at all) to 7 (extremely restricted).

**Fig 7:**
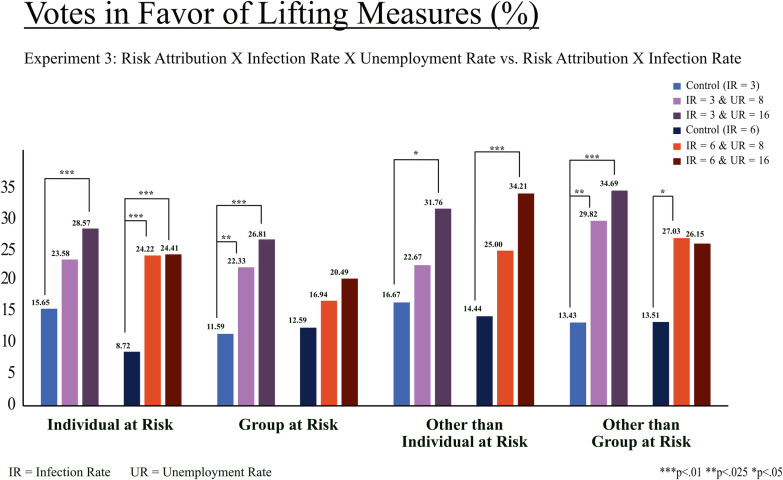
Votes in favor of lifting COVID-19 measures (Experiment 3). Note: Proportion of votes in favor of lifting measures, considering the presented trade-off. Proportion tests between groups (within and between Infection Rate and Unemployment Rate) show no significant differences. In addition, proportion tests between Experiment 1 and 3 have been carried out. Asterisks indicate significant differences between the treatment group in Experiment 1 and the indicated treatment group in Experiment 3.

Due to the experimental design of Experiment 3, we are able to analyze any differences when compared to Experiment 1. That is, we analyze the effect of communicating higher unemployment rates and infection rates jointly as opposed to only communicating increased infection rates. We conducted proportion tests to compare the conditions in Experiment 3, where infection rates and unemployment rates are presented jointly, to the corresponding conditions in Experiment 1 (i.e., control). We find that votes in favor of lifting the measures in most conditions are higher (Fig 7).

#### Risk attribution: individual at risk.

In the scenario that risk attribution is at individual level, significantly more people vote in favor of lifting the measures when unemployment rates increase to a high level (28.57% vs 15.65%, *z* =  2.287, *p* < .01; Fig 7). The proportion of votes for lifting the measures is also significantly higher when the infection rate increases to six (for UR =  8: 24.22% vs 8.72%, *z* =  3.517, *p* < .01; for UR =  16%: 24.41% vs 8.72%, *z* =  3.548, *p* < .01).

#### Risk attribution: group at risk.

 Even when participants are primed that their group (i.e., themselves and their closest relatives) are susceptible to infection (i.e., at risk), votes for lifting the measures are higher compared to Experiment 1 (for UR =  8: 22.33% vs 11.59%, *z* =  2.239, *p* = *.*025; for UR =  16%: 26.81% vs 11.59%, *z* =  3.209, *p* = .001), however, not at the higher level of increased infection rate (i.e., not for IR =  6).

#### Risk attribution: other than individual at risk.

 When people other than the individual are at risk, significantly more participants vote for lifting the restrictions on private and public life compared to Experiment 1 when unemployment rates highly increase (for IR =  3: 31.76% vs 16.67%, *z* =  2.121, *p* = .034; for IR =  6: 34.21% vs 14.45%, *z* =  2.993, *p* = .003), but there is no uplift in votes for the lower level of increased unemployment rate.

#### Risk attribution: other than group at risk.

Lastly, priming for risk attribution to people outside of the group leads to a higher proportion of votes in favor of lifting the measures compared to Experiment 1 (for IR =  3 & UR =  8: 29.82% vs 13.43%, *z* =  2.235, *p* = .025; for IR =  3 & UR =  16: 34.69% vs 13.43%, *z* =  2.712, *p* = .007; for IR =  6 & UR =  8: 27.03% vs 13.51%, *z* =  2.045, *p* = .041), exce*p*t for when both IR and UR are at the higher level.

## Discussion

The COVID-19 pandemic posed many challenges worldwide as governments needed to balance health safety against the consequences of disrupting public and private life. Strategies to minimize infections restricted civil liberties and hurt the economy. Moreover, education and working conditions were crucially affected [32]. Given the critical trade-off, the acceptance of the restrictions was paramount as lifting measures prematurely can lead to a quick resurgence of the disease [33]. In Germany, the government communicated the severe effects of COVID-19 on health safety to maintain the acceptance of the measures despite the expected economic downturn [34]. In fact, crisis management imposed by governments relies on the acceptance and compliance of individuals and is, thus, imperative.

First, our results show that indeed individuals viewed the trade-off as very strong. Participants specifically perceived the presented consequences of COVID-19 as threatening to them and others but simultaneously felt that maintaining the COVID-19 measures in place was a substantial restriction of freedom to themselves and others. Nonetheless, the results indicate that governmental measures aimed at reducing virus transmissions, securing national health systems, and limiting virus-related death victims were highly accepted by individuals in Germany. Due to our experimental design, we can offer a behavioral distribution of acceptance of the measures given specific communication strategies.

From a moral point of view, we observe that individuals act ethically. They tend to accept individual harm (i.e., restrictions on private and public life) to prevent increased societal harm (i.e., infections, deaths). Moreover, when communication focuses on health threats like infection and death rates, decision behavior is generally robust to different perceptions of risk attribution (Experiments 1 and 2). That is, acceptance levels do not significantly change based on who is endangered by their decision, except in Experiment 2 where more people voted to lift the measures when their own group was immune than when only their own person was immune. However, additionally communicating economic consequences (Experiment 3), measured by higher unemployment rates as a result of maintaining COVID-19 measures, significantly reduces acceptance. In addition, we find that when both health and economic consequences are communicated (Experiment 3), attributing risk to one’s own group relative to others increases acceptance, so significantly fewer participants vote to lift COVID-19 measures when one’s own group is at greater risk relative to others. Moreover, we find a positive effect of the level of unemployment rate on the decision to lift restrictive measures.

We lastly compared Experiment 3 to Experiment 1: When unemployment rates increase, especially at a higher level, votes to lift measures are higher than in scenarios without communication of economic consequences (Experiment 1). However, when health consequences are serious, that is, a higher increase in infection rates and when the risk of infection is attributed to the participant’s group, information about unemployment rates does not significantly affect votes for lifting the measures. This indicates that risk attribution to one’s group can overweigh negative economic consequences when communicated together.

Our results extend the literature stream on acceptance of COVID-19 measures. Between March 2020 and March 2021, several studies in different countries and at different stages of the pandemic examined public preferences for different COVID-19 measures and various factors influencing choice behavior, including health, economic and educational impacts, and risk perceptions (see the literature table in Supporting Information F). In general, these studies show a significant support for restrictive COVID-19 measures to mitigate health risks, even despite economic disadvantages [8–13] or educational impairments [8,13]. In studies from the USA [9,11], Australia [10], the Netherlands [8] and Portugal [13], health-related factors consistently and most strongly influence public support for COVID-19 measures, but economic factors also affect decisions. Only in Germany did a decline in individual income have the highest impact on choice behavior [12]. In this study, we extend previous research and demonstrate that the majority chooses to maintain all restrictive measures on private and public life, when only health-related consequences are communicated. Communication strategies that incorporate both health-related and economic-related consequences lead to more individuals voting to lift restrictions.

With the exception of [35], who conducted an online experiment, all previous studies used discrete choice experiments. This study reduces the hypothetical bias of discrete choice experiments by using a referendum setting to examine the acceptance of restrictive COVID-19 measures. While discrete choice experiments assess preferences and relative importances between different scenarios including a wide range of attributes, the referendum setting simplifies the decision-making process to a more realistic scenario [14]. Specifically, we focus on the influence of different communication foci (health-related vs. economic) on individuals’ choice behavior during the presented social dilemma. We contribute to this literature by measuring voting behavior in a referendum setting that mimics a real-world democratic vote during the first phase of the pandemic and while all restrictions were active in Germany. Moreover, the trade-off scenarios in this study incorporate real COVID-19 metrics communicated in the media. Our results highlight the importance and influence of different communication foci on individual acceptance of restrictive COVID-19 measures. Last, we also contribute to the current literature on behavioral studies of COVID-19 acceptance by also including the role of risk attribution (i.e., who is at risk).

Furthermore, we advance the understanding of communication strategies in the context of the COVID-19 pandemic, contributing to the social dilemma theory [e.g., 1]. In relation to the cognitive appraisal of crisis scenarios [25], our findings indicate that in the early phase of a pandemic, communicating health factors (i.e., both infection and death rates) effectively signals a threat to well-being and encourages prosocial behavior. Communicating infection rates or death rates does not lead to different voting outcomes. However, our results suggest that adding economic consequences (i.e., unemployment rates) into communication can undermine the positive effect of health-related consequences on the acceptance of restrictive measures, which may be perceived as more abstract risks. This joint communication of health and economic consequences motivated more individuals to vote in favor of lifting the restrictive measures.

Our results demonstrate that individuals are susceptible to communication strategies with different foci when faced with a social dilemma like a pandemic. We show how both communication strategies and government support programs that mitigate economic threats can increase the acceptance of pandemic measures, especially at the onset of a pandemic. The COVID-19 pandemic showed a dramatic increase in national debt, which increases the debt burden for many years and can push the economic system towards long-term distress. Thus, governments have limited time to maintain measures during a pandemic as each intensification of said measures increases the economic burden and reduces individuals’ will to comply. In fact, tens of thousands of people in Germany and other European countries protested against COVID-19 measures in 2021 [e.g., 36], and against both measures and vaccination policies in 2022 [e.g., 37].

Moreover, risk attribution has significant implications when the population faces economic downturns. While our results show that people are generally insensitive to changes in risk attribution at the beginning of a pandemic, increased immunization by infection or vaccination and noticeably bleak economic prospects may reduce acceptance of measures. This comes at the cost of at-risk groups. Thus, on the one hand policymakers must solve the social dilemma of preventing a significant economic downturn while compromising civil liberties, and securing the healthcare system while saving the lives of at-risk groups on the other.

While at this time, COVID-19 measures have been lifted, a recent study estimates that there is a 38% chance of experiencing a pandemic with similar impact in our lifetime and this risk can increase threefold in the coming decades [38]. Managing strategies to fight pandemics raises ethical questions for governments and remains a challenge given the threat of future pandemics.

This research has some limitations. First, our results represent the decision behavior of German participants which may differ in both perception and behavior from populations in other countries. However, the measures studied in our experiments are similar to those adopted in over 150 countries around the world [39]. Second, we studied voting behavior in a controlled online experiment. However, all three experiments were conducted during the first phase of the pandemic, when all restrictive COVID-19 measures were still in place and virus transmissions were a substantial threat to all individuals, increasing external validity. Third, at the time that we conducted the experiments the perceived risk of infection was high and the level of temporal immunization after infection was low. This may reduce generalizability to later stages of a pandemic, when perceived risk of infection is lower and more individuals have recovered from a COVID-19 infection. Yet, effective crisis communication in the early stages of a pandemic is critical to prevent deaths and negative economic consequences.

## Supporting information

S1 File Trading-Off Health Safety, Civil Liberties, and Unemployment Based on Communication Strategies: The Social Dilemma in Fighting Pandemics. (PDF)
