# Supplementary material for: Trading-off health safety, civil liberties, and unemployment based on communication strategies: the social dilemma in fighting pandemics
Source: PLoS One. 2025 Mar 3;20(3):e0318541. doi: 10.1371/journal.pone.0318541 (PMC11875376; doi:10.1371/journal.pone.0318541)
Supplement: S1 File — (PDF) [file pone.0318541.s001.pdf]

Supporting Information for

Trading-Off Health Safety, Civil Liberties, and Unemployment Based on Communication Strategies: The Social Dilemma in Fighting the COVID-19 Pandemic

Contents

A Experimental Stimuli: Experiments 1-3

B Descriptive Statistics

C Additional results Experiment 1

D Additional results Experiment 2

E Additional results Experiment 3

F Literature Overview

Supporting Information A. *EXPERIMENTAL STIMULI (TRANSLATED TO ENGLISH FROM GERMAN)*

Procedure and Stimuli.

Participants are randomly assigned to experimental conditions in each experiment. All participants are shown an introduction at the beginning of the experiment with information about the current COVID-19 situation and the active measures (Figure S1).

Figure S1: Introduction

Please read the following text carefully and imagine this initial situation.

Due to the spread of the Covid-19 virus and the threat of a state of emergency in Germany, the government issued a <u>government decree</u> imposing a wide-ranging ban on gatherings and meetings of more than 2 people as a measure <u>to fight the spread of the virus.</u>

The following areas are affected:

- **<u>Reduction of contacts</u> to an absolutely necessary minimum with other people (outside the members of the own household)**
- **<u>Minimum distance</u> of at least 1.5 m in public**
- **<u>Staying in the public space is allowed only alone or with another person</u> (outside the members of the own household)**
- **<u>Necessary routes in public space</u> are allowed**

  Getting to work, to emergency care, grocery shopping, doctor's appointments, attending meetings, required appointments, and exams, helping others, or individual sports and exercise in the fresh air remain possible.
- **<u>Catering establishments</u> are closed (Except: Delivery and collection of takeaway food for consumption at home)**
- **Grocery stores (e.g., supermarkets) are open**

Next, participants are shown risk attribution stimuli, i.e., they are informed whether they (and closest relatives) are immune or susceptible to an infection (Table S1), depending on the experimental condition.

Table S1: Risk Attribution Stimuli

**Individual at Risk**	Please imagine that you have not been infected before. Therefore, you are basically susceptible to corona virus infection (Covid-19).
**Group at Risk**	Please imagine that you and your closest relatives have not been infected so far. Therefore, you are basically susceptible to corona virus infection (Covid-19), which means that you and your closest relatives are not immune.
**Other than Individual at Risk**	Please imagine that you had already been infected and recovered. Therefore, you are no longer susceptible to corona virus infection (Covid-19), i.e., you are immune.
**Other than Group at Risk**	Please imagine that you and your closest relatives had already become infected and recovered. Therefore, you are no longer susceptible to corona virus infection (Covid-19), i.e., you and your closest relatives are immune.

Third, baseline levels of infection rate (Experiment 1), death rate within risk groups (Experiment 2), and infection rate and unemployment rate (Experiment 3) under the active COVID-19 measures are presented (Table S2). Participants in Experiment 2 were additionally shown a definition of risk groups (Figure S2) before the baseline of death rate was presented.

Table S2: Baseline levels

**Infection Rate**	Under the described restrictive COVID-19 measures, the following evolution of Covid-19 spread is observed: The infection rate decreases and is 1.5, i.e., one infected person statistically infects 1.5 people.
**Death Rate**	Under the described restrictive COVID-19 measures, the following death rates are observed: The death rate because of Covid-19 infection for the…<br>- Non-risk group is 2%, i.e., 2 out of 100 diseased persons die<br>- Risk group is at 7.5%, i.e., 7.5 out of 100 diseased persons die
**Infection Rate & Unemployment Rate**	Under the described restrictive COVID-19 measures, the following development is observed: Spread of the virus:<br>The infection rate decreases and is 1.5, i.e., one infected person statistically infects 1.5 people.<br>Labor market development:<br>The unemployment rate increases by 4 percentage points, i.e., on average 4 out of 100 people lose their jobs due to the corona crisis.

Figure S2: Definition of Risk Groups

Please read the following text on risk groups published by the Robert-Koch-Institute carefully:

Groups of people at increased risk for severe disease progression with Covid-19:

Elderly people

The risk of severe disease increases steadily with age from age 50 to 60. Older people can become more seriously ill after an infection due to their less responsive immune system.

People with various underlying diseases and people with disabilities

E.g., cardiovascular diseases, diabetes, diseases of the respiratory system, liver and kidney, and cancers appear to increase the risk of severe disease progression regardless of age.

Elderly people with pre-existing underlying diseases

The risk of severe disease progression is higher when multiple underlying diseases are present.

People with suppressed immune system

E.g., due to an illness or taking medication that is associated with an immune deficiency or suppresses the immune defense, there is a higher risk.

Last, participants are presented with a referendum decision to lift or keep the restrictive COVID-19 measures. Each decision option has its consequences, which are also presented according to the experimental condition. Figures 3-8 show the referendum decisions corresponding to the manipulations of infection rate, death rate, and unemployment rate (see also Table 1 for an overview of studies and manipulations).

Figure S3: Referendum Experiment 1 (IR = 3)

Now imagine having to decide in a legally binding referendum whether to continue the previously described measures to control the spread of the corona virus (Covid-19) for another 30 days or to repeal them with immediate effect.

Your vote decides the outcome of the referendum.

Both outcomes of the referendum have consequences for other people.

Which scenario do you choose? Please make a choice.

	**My choice**
**Scenario: Lifting Measures**<br><br>Removal of restrictions<br>on private and public<br>life<br>&<br>3 people are infected<br>per infected person	☐
**Scenario: Keeping Measures**<br><br>Maintaining restrictions<br>on private and public<br>life<br>&<br>1.5 people are infected<br>per infected person	☐

Figure S4: Referendum Experiment 1 (IR = 6)

Now imagine having to decide in a legally binding referendum whether to continue the previously described measures to control the spread of the corona virus (Covid-19) for another 30 days or to repeal them with immediate effect.

Your vote decides the outcome of the referendum.

Both outcomes of the referendum have consequences for other people.

Which scenario do you choose? Please make a choice.

	**My choice**
**Scenario: Lifting Measures**<br><br>Removal of restrictions on private and public life<br>&<br>6 people are infected per infected person	☐
**Scenario: Keeping Measures**<br><br>Maintaining restrictions on private and public life<br>&<br>1.5 people are infected per infected person	☐

Figure S5: Referendum Experiment 2 (DR = 15)

Now imagine having to decide in a legally binding referendum whether to continue the previously described measures to control the spread of the corona virus (Covid-19) for another 30 days or to repeal them with immediate effect.

Your vote decides the outcome of the referendum.

Both outcomes of the referendum have consequences for other people.

Which scenario do you choose? Please make a choice.

My choice

Scenario: Lifting Measures

Removal of restrictions
on private and public
life
&
15 out of 100 high-risk patients
die as a result of COVID-19

☐

Scenario: Keeping Measures

Maintaining restrictions
on private and public
life
&
7.5 out of 100 high-risk patients
die as a result of COVID-19

☐

Figure S6: Referendum Experiment 2 (DR = 25)

Now imagine having to decide in a legally binding referendum whether to continue the previously described measures to control the spread of the corona virus (Covid-19) for another 30 days or to repeal them with immediate effect.

Your vote decides the outcome of the referendum.

Both outcomes of the referendum have consequences for other people.

Which scenario do you choose? Please make a choice.

	**My choice**
**Scenario: Lifting Measures**<br><br>Removal of restrictions<br>on private and public<br>life<br>&<br>25 out of 100 high-risk patients<br>die as a result of COVID-19	☐
**Scenario: Keeping Measures**<br><br>Maintaining restrictions<br>on private and public<br>life<br>&<br>7.5 out of 100 high-risk patients<br>die as a result of COVID-19	☐

Figure S7: Referendum Experiment 3 (IR = 3, UR = 8)

Now imagine having to decide in a legally binding referendum whether to continue the previously described measures to control the spread of the corona virus (Covid-19) for another 30 days or to repeal them with immediate effect.

Your vote decides the outcome of the referendum.

Both outcomes of the referendum have consequences for other people.

Which scenario do you choose? Please make a choice.

	**My choice**
**Scenario: Lifting Measures**<br><br>Removal of restrictions on private and public life<br>&<br>4 out of 100 persons lose their job due to the COVID-19 crisis<br>&<br>3 people are infected per infected person	☐
**Scenario: Keeping Measures**<br><br>Maintaining restrictions on private and public life<br>&<br>8 out of 100 persons lose their job due to the COVID-19 crisis<br>&<br>1.5 people are infected per infected person	☐

Figure S8: Referendum Experiment 3 (IR = 3, UR = 16)

Now imagine having to decide in a legally binding referendum whether to continue the previously described measures to control the spread of the corona virus (Covid-19) for another 30 days or to repeal them with immediate effect.

Your vote decides the outcome of the referendum.

Both outcomes of the referendum have consequences for other people.

Which scenario do you choose? Please make a choice.

	**My choice**
**Scenario: Lifting Measures**<br><br>Removal of restrictions<br>on private and public<br>life<br>&<br>4 out of 100 persons lose<br>their job due to the COVID-19 crisis<br>&<br>3 people are infected<br>per infected person	☐
**Scenario: Keeping Measures**<br><br>Maintaining restrictions<br>on private and public<br>life<br>&<br>16 out of 100 persons lose<br>their job due to the COVID-19 crisis<br>&<br>1.5 people are infected<br>per infected person	☐

Figure S9: Referendum Experiment 3 (IR = 6, UR = 8)

Now imagine having to decide in a legally binding referendum whether to continue the previously described measures to control the spread of the corona virus (Covid-19) for another 30 days or to repeal them with immediate effect.

Your vote decides the outcome of the referendum.

Both outcomes of the referendum have consequences for other people.

Which scenario do you choose? Please make a choice.

	**My choice**
**Scenario: Lifting Measures**<br><br>Removal of restrictions<br>on private and public<br>life<br>&<br>4 out of 100 persons lose<br>their job due to the COVID-19 crisis<br>&<br>6 people are infected<br>per infected person	☐
**Scenario: Keeping Measures**<br><br>Maintaining restrictions<br>on private and public<br>life<br>&<br>8 out of 100 persons lose<br>their job due to the COVID-19 crisis<br>&<br>1.5 people are infected<br>per infected person	☐

Figure S10: Referendum Experiment 3 (IR = 6, UR = 16)

Now imagine having to decide in a legally binding referendum whether to continue the previously described measures to control the spread of the corona virus (Covid-19) for another 30 days or to repeal them with immediate effect.

Your vote decides the outcome of the referendum.

Both outcomes of the referendum have consequences for other people.

Which scenario do you choose? Please make a choice.

My choice

Scenario: Lifting Measures

Removal of restrictions
on private and public
life
&
4 out of 100 persons lose
their job due to the COVID-19 crisis
&
6 people are infected
per infected person

☐

Scenario: Keeping Measures

Maintaining restrictions
on private and public
life
&
16 out of 100 persons lose
their job due to the COVID-19 crisis
&
1.5 people are infected
per infected person

☐

Supporting Information B. *DESCRIPTIVE STATISTICS AND MEASUREMENTS*

Table S3: Descriptive Statistics

	Experiment 1<br>N = 866		Experiment 2<br>N = 817		Experiment 3<br>N = 1,564	
	Absolute Number	Percentage	Absolute Number	Percentage	Absolute Number	Percentage
Gender						
Female	429	49.54	393	48.10	779	49.81
Male	436	50.35	422	51.65	784	50.13
Other	1	00.12	2	00.24	1	00.06
Average Age (in years)	46.97		46.49		46.05	

Table S4: Measurements

	Experiment 1<br>N = 866		Experiment 2<br>N = 817		Experiment 3<br>N = 1,564	
	Mean	SD	Mean	SD	Mean	SD
**Perceived Threat**	5.52	1.41	5.55	1.35	5.34	1.45
Cronbach's α = .93						
If I vote to lift the measures:<br>I put others at risk<br>others are put at risk<br>I will harm others<br>others are harmed						
**Perceived Restriction**	5.70	1.36	5.63	1.31	5.66	1.26
Cronbach's α = .93						
If I vote to keep the measures:<br>I am restricted in my freedom<br>others are restricted in their freedom<br>public and private life is restricted for me<br>public and private life is restricted for others						
**Decision Difficulty**	2.41	1.62	2.45	1.66	3.07	1.83
How difficult or easy was it for you to decide on a scenario in the referendum?						
**Decision Confidence**	5.51	1.57	5.49	1.58	5.10	1.61
How sure or uncertain are you about your decision in the referendum?						
**Expected Return to Daily Life**	4.80	1.44	4.80	1.39	4.88	1.40
When do you think you will be able to return to your usual routine?<br>1 = in the next two weeks; 2 = next month; 3 = in the next two months; 4 = in the next three months;<br>5 = in the next six months; 6 = next year; 7 = in over one year						
**Current Restriction of Daily Life**	4.61	1.63	4.62	1.64	4.70	1.58
To what extent do you feel restricted in your daily life?						

Supporting Information C. *ADDITIONAL RESULTS EXPERIMENT 1*

Table S5: Logistic Regression Experiment 1

	**Model 1**			**Model 2**		
	Odds Ratio	se	*p*	Odds Ratio	se	*p*
**Risk Attribution (RA)**				.94	.133	.674
Individual at Risk (1)	.88	.269	.688			
Group at Risk (2)	.88	.271	.671			
Other than Individual at Risk (3)	1.18	.392	.616			
Other than Group at Risk (4)	*serves as reference group*			*serves as reference group*		
**Infection Rate (IR)** (0=3, 1=6)	.80	.164	.286	1.01	.497	.989
**Interaction**						
**RA X IR**						
RA=1 : IR=1				.51	.315	.277
RA=2 : IR=0				.75	.246	.382
RA=2 : IR=1				.82	.420	.696
RA=3 : IR=0				1.21	.503	.639
RA=3 : IR=1				1.02	.484	.970
**Constant**	.17	.046	.000	.20	.064	.000
N	866			866		

Note: The outcome variable is binary, where an outcome of 1 indicates the decision to lift measures, while 0 indicates maintaining them. The odds ratios represent the probability of deciding to lift measures compared to the reference group or baseline condition. For the "Risk Attribution" (RA) variable, the reference group is "Other than Group at Risk." For the "Infection Rate" (IR), the variable is coded as 1 for a higher infection rate (IR=6) and 0 for the baseline lower infection rate (IR=3). The interaction term “RA X IR” assess the interaction effects between risk attribution and infection rate on the decision to lift measures.

Supporting Information D. *ADDITIONAL RESULTS EXPERIMENT 2*

Table S6: Logistic Regression Experiment 2

	Model 1			Model 2		
	Odds Ratio	se	*p*	Odds Ratio	se	*p*
**Risk Attribution (RA)**				1.21	.166	.173
Individual at Risk (1)	.88	.270	.676			
Group at Risk (2)	.97	.297	.912			
Other than Individual at Risk (3)	.66	.238	.250			
Other than Group at Risk (4)	*serves as reference group*			*serves as reference group*		
**Death Rate (DR)** (0=15, 1=25)	.87	.171	.466	.44	.230	.116
**Interaction**						
**RA X DR**						
RA=1 : DR=1				2.50	1.56	.142
RA=2 : DR=0				1.16	.346	.610
RA=2 : DR=1				1.73	.947	.313
RA=3 : DR=0				.38	.178	.039
RA=3 : DR=1				1.65	.885	.349
**Constant**	.21	.060	.000	.14	.046	.000
N	817			817		

Note: The outcome variable is binary, where an outcome of 1 indicates the decision to lift measures, while 0 indicates maintaining them. The odds ratios reflect the probability of deciding to lift measures compared to the reference group or baseline condition. The "Risk Attribution" (RA) variable's reference group is "Other than Group at Risk." For the "Death Rate" (DR) variable, a value of 1 corresponds to a higher death rate (DR=25), with the baseline being a lower death rate (DR=15). The interaction term “RA X DR” assess the interaction effects between risk attribution and death rate on the decision to lift measures.

Supporting Information E. *ADDITIONAL RESULTS EXPERIMENT 3*

Table S7: Logistic Regression Experiment 3

	Model 1			Model 2			Model 3		
	Odds Ratio	se	*p*	Odds Ratio	se	*p*	Odds Ratio	se	*p*
**Risk Attribution (RA)**									
Individual at Risk (1)	.79	.193	.338	.74	.188	.235	.81	.142	.229
Group at Risk (2)	.61	.156	.053	.68	.176	.139	.66	.119	.022
Other than Individual at Risk (3)	.80	.212	.397	.79	.216	.390	.95	.178	.799
Other than Group at Risk (4)	*serves as reference group*			*serves as reference group*			*serves as reference group*		
**Unemployment Rate (UR)** (0=8, 1=16)	.108	.305	.783	1.25	.147	.054	1.34	.223	.076
**Infection Rate (IR)** (0=3, 1=6)	.86	.101	.211	.77	.217	.349	.93	.158	.653
**Interactions**									
**RA X UR**									
RA=1 : UR=1	1.06	.370	.867						
RA=2 : UR=1	1.19	.428	.625						
RA=3 : UR=1	1.43	.525	.344						
**RA X IR**									
RA=1 : IR=1				1.19	.416	.623			
RA=2 : IR=1				.920	.331	.817			
RA=3 : IR=1				1.44	.540	.330			
**IR X UR**									
IR=1: UR=1							.87	.204	.552
**Constant**	.43	.087	.000	.42	.092	.000	.38	.068	.000
N	1,564			1,564			1,564		

Note: The outcome variable is binary, where an outcome of 1 indicates the decision to lift measures, while 0 indicates maintaining them. The odds ratios represent the probability of deciding to lift measures compared to the reference group or baseline condition. For the "Risk Attribution" (RA) variable, the reference group is "Other than Group at Risk." For the binary variables "Infection Rate" and "Unemployment Rate," a value of 1 indicates the higher level (IR=6 and UR=16, respectively), with the baseline being IR=3 and UR=8. The interaction terms “RA X IR” ,“RA X IR”, “IR X UR” assess the interaction effects between these variables on the decision to lift measures.

Supporting Information F. *Literature Overview*

**Data Collection**	March – April 2020	*April – May 2020*	April 2020	May 2020	July – August 2020	August 2020	October – November 2020	January – March 2021
**Study**	Chmel et al., (2021)	*This Study*	Chorus et al. (2020)	Reed et al. (2020)	Manipis et al. (2020)	Li et al. (2021)	Mühlbacher et al. (2022)	Filipe et al. (2022)
**Country**	Russia	*Germany*	Netherlands	USA	Australia	USA	Germany	Portugal
**Method**	Online Experiment	*Online Experiment*<br>*(Referendum)*	Discrete Choice Experiment	Discrete Choice Experiment	Discrete Choice Experiment	Discrete Choice Experiment	Discrete Choice Experiment	Discrete Choice Experiment
**Experimental Factors / Attributes**	• Risk Severity<br>• Object at Risk (protect myself vs. protect others)	• *Infection Rate*<br>• *Death Rate*<br>• *Unemployment Rate*<br>• *Risk Attribution*	• Deaths<br>• Lasting physical injuries<br>• Lasting mental injuries<br>• Educational impairment<br>• Income losses (poverty)<br>• Additional Household Tax<br>• Work pressure in the health sector	• Infections<br>• Duration of restrictions on non-essential businesses<br>• Poverty level<br>• Time for economic recovery	• Deaths<br>• Infections<br>• Job Losses<br>• Government Expenditure<br>• Additional Personal Tax<br>• Restriction Level<br>• Duration of Restrictions<br>• Tracking	• Increase in Infections<br>• Increase in Unemployment Insurance Claims<br>• Length of restriction (Stay-at-home order)<br>• Mask mandate<br>• School Opening Probability	• Deaths<br>• Income lost<br>• Economic Performance (decline in GDP)<br>• Individual Risk of Infection<br>• Curfews<br>• Contact Restrictions<br>• Closures<br>• Mask Obligation<br>• Transmission of Personal Data	• Deaths<br>• Income lost<br>• Educational impairment<br>• Type of Restriction<br>• Poverty Level
**Health-Related**	**X**	**X**	**X**	**X**	**X**	**X**	**X**	**X**
**Economic**	**n.a.**	**X**	**X**	**X**	**X**	**X**	**X**	**X**
**Educational**	**n.a.**	**n.a.**	**X**	**n.a.**	**n.a.**	**X**	**n.a.**	**X**

**Risk-related (perceptions / attribution)**	**X**	**X**	**n.a.**	**n.a.**	**n.a.**	**n.a.**	**X**	**n.a.**
**Type of Life Restriction**	**n.a.**	**X**	**n.a.**	**X**	**X**	**X**	**X**	**X**
**Outcome Measure**	• Willingness to sacrifice individual rights (5-point scale)<br>• Support of COVID-19 measures (5-point scale)<br>• Support for criminal liability for violation of quarantine (5-point scale)	*Lift COVID-19 measures or Maintain COVID-19 measures*	Choice Behavior	Choice Behavior<br><br>(Willingness to accept COVID-19 infection risks for earlier lifting of restrictions)	Choice Behavior	Choice Behavior<br><br>(Willingness to stay at home / preferences regarding length of potential lockdown)	Choice Behavior (Willingness to accept COVID-19 measures)	Choice Behavior
**Main Results**	**Higher Risks (Mortality Rate)** leads to higher support compared to Lower Risks (Recovery Rate).<br><br>No significant differences between losses for others (protect myself) and individual losses (protect others) framing	***Majority** chooses to **keep the restrictions** on private and public life.*<br><br>***Significantly more individuals choose lifting restrictions** when **economic consequences** are communicated.*	Majority is willing to trade-off effects of lock-down (economic and educational) for **health-related** effects	**Majority preferred** to avoid COVID-19 risks (**infections**) and delay reopening nonessential businesses	**Highest preferences** for policies that avoid high infection-related **deaths**	Broad support for statewide mask mandate. **To lower new infections**, willingness to stay home is highest (five and a half weeks).	**Individual income** decreases have the highest impact on **choice decisions**	**Highest preferences** for avoiding **deaths**
